# Effects of Dietary Hempseed or Camelina Cakes on Fatty Acid Composition of Quail Meat

**DOI:** 10.3390/life14010053

**Published:** 2023-12-28

**Authors:** Robertas Juodka, Rasa Nainienė, Artūras Šiukščius, Raimondas Leikus, Giedrius Šarauskas

**Affiliations:** 1 Department of Ecology, Animal Science Institute, Lithuanian University of Health Sciences, R. Zebenkos 12, 82317 Baisogala, Lithuania; 2 Department of Animal Breeding and Reproduction, Animal Science Institute, Lithuanian University of Health Sciences, R. Zebenkos 12, 82317 Baisogala, Lithuania; arturas.siukscius@lsmu.lt (A.Š.); giedrius.sarauskas@lsmu.lt (G.Š.); 3 Department of Animal Feeding and Feedstuffs, Animal Science Institute, Lithuanian University of Health Sciences, R. Zebenkos 12, 82317 Baisogala, Lithuania; raimondas.leikus@lsmu.lt

**Keywords:** quail, oilseed by-products, n-3 fatty acids, meat, health

## Abstract

The purpose of the study was to investigate the effects of dietary hempseed or camelina cakes on the fatty acid profiles of intramuscular fat in quail. A total of 189 one-day-old quail were allocated to three dietary treatment groups. The diet of the control (C) group was supplemented with 10% rapeseed cake, whereas the rapeseed in experimental 1 (HE) and experimental 2 (CA) groups was replaced by, respectively, hempseed cake and camelina cake in the same proportions. The length of the study was 42 days. Dietary enrichment with camelina cake increased the α-linolenic fatty acid (ALA) content in the meat of CA group 2.5 times (*p* < 0.01). The muscle tissues of CA contained 3.4–3 times more eicosapentaenoic acid (*p* < 0.01), 1.2 times more docosapentaenoic acid (*p* < 0.05–*p* < 0.01) and 1.3 times more docosahexaenoic acid (*p* < 0.01) and, thus, demonstrated the increase in total long chain (LC) n-3 polyunsaturated fatty acids (PUFA) (*p* < 0.01) and total n-3 PUFA (*p* < 0.01) compared with the C group. The ALA and total n-3 PUFA content in the breast and leg meat of HE-treated quail were, respectively, 1.3 and 1.1 times higher (*p* < 0.01) than in the C group but the accumulation was lower compared to the CA group. The content of γ-linolenic acid was found to be 1.21–1.31 times higher in HE quail meat (*p* < 0.01). However, hempseed cake supplementation had a negative effect on growth performance. The supplementation of quail feed with camelina or hempseed cakes resulted in the production of healthy meat with an increased n-3 PUFA content.

## 1. Introduction

Consumers give preference to poultry meat due to its high quality, nutritive value and lower costliness compared to other meats. Epidemiological studies conducted in diverse populations with different food preferences and nutritional habits established a relation between the consumption of poultry in a balanced diet and good health [1].

Broiler chickens are the most produced poultry in the world. In 2020, 33 billion broil-er chickens were raised [2]. It is predicted that animal protein consumption will continue to increase in low- and middle-income countries due to population and income growth. Meanwhile, it is expected that in high-income countries the consumption of poultry meat will stabilize or decrease [2]. In the aging populations of the latter countries, consumer decisions are increasingly influenced by the health effects of consumed products. Greater protein source diversity is also desirable and the impact of production on the environment is being taken into account.

One of such alternatives that could occupy a specific niche is quail meat. An estimated 1.4 billion quail are farmed annually, which accounts for 0.2% of the total poultry production [3,4]. Over 80% of quail are grown in China. More than 100 million quail are raised annually in the European Union [4]. Compared to other bird species, quail production is distinguished by the possibility to grow large quail numbers in small facilities, moreover, quail are characterized by early sexual maturity, shorter generation interval (2 to 5 generations per year can be achieved), are known for their longevity, heat tolerance, resistance to diseases and low production cost [3,5,6]. In many countries, among consumers, the opinion has been formed that quail meat is more valuable than that of chickens grown under intensive poultry farming and stands out as being more natural and healthier [5].

Studies show that quail meat contains more fat than chicken, but the composition of fatty acids in quail is more favorable to human health [7,8,9,10,11,12]. The content of harmful to human health saturated fatty acids (SFA) in quail breast is lower [12,13,14,15] and that of favorable α-linolenic fatty acid (ALA) higher compared to chicken [12,13,14,15]. The n-6/n-3 ratio of polyunsaturated fatty acids (PUFA), which important in human nutrition, in the breast meat of quail and chicken is similar (9.54–22.10 vs. 13.77–18.2) [12,13,14,15], but higher than that to be found in a healthy diet [16].

Research has established a direct positive correlation between the amount of n-3 polyunsaturated fatty acids (PUFA) in feed and n-3 PUFA accumulation in the body tissues [17]. Therefore, there arises a possibility to manipulate the fatty acid composition of birds’ muscles through the composition of the feed and, thus, generate consumer health beneficial food.

In order to increase the sustainability of poultry production by implementing the Sustainable Development Goals of the United Nations and EU Farm to Fork Strategy, it is recommended to reduce dependence on imported feed raw materials, for example, soya; shorten supply chains; and look for alternative protein and oilseed crops adapted to local conditions.

Camelina (*Camelina sativa* (L.) Crantz) [18] and hemp (*Cannabis sativa* L.) [19] has recently become a matter of interest in agriculture and industry. These underexploited crops are naturally resistant to pests and diseases, require less mineral fertilization and water [20,21] and are considered more sustainable than other cultures [22] satisfying the goals the EU Green Deal for reduced chemical input use by 2030. Moreover, camelina and hemp are multipurpose crops widely used in food, feed, chemical and energy industries. After extracting oil from their seeds, considerable amounts of cake containing valuable nutrients are formed, which can be used for bird feeding. The protein content in camelina cake amounts to 33.1–38.42% [23,24], and that of fat to 10.52–13.9% [23,24], with as much as 25.88–31.50% ALA [25,26,27,28] and 26.72–33.36% total n-3 PUFA [25,26,27].

Meanwhile, hempseed cake contains 24.8–34.3% protein [29,30,31], 8.7–12.7% fat [29,30], 12.82–19.1% ALA [26,29,32] and 14.62–19.1% total n-3 PUFA [26,29,32].

Research shows that supplementation of the chicken diet with camelina cake increases the accumulation of n-3 PUFA in their muscles. In breast muscles, ALA was found to be 1.34 to 3.90 times higher, the total n-3 PUFA was 1.32 to 3.23 times higher and the n-6/n-3 PUFA ratio was decreased from 1.32 to 2.83 times [7,10,27,33,34]. In thigh muscles, ALA increased from 1.32 to 4.37 times and the total n-3 PUFA from 1.74 to 3.73 times. Meanwhile, the n-6/n-3 PUFA ratio decreased from 1.78 to 2.89 times [7,27].

Jing et al. [35] indicated that the supplementation of the chicken diet with hemp oil resulted in 2.46–5.45 times higher ALA content, 3.82–14.76 times higher total n-3 PUFA content and 2.25–3.25 times higher long-chain (LC) n-3 PUFA content in the meat.

So far, we have not been able to find any scientific research aimed at evaluating the changes in the fatty acid content of quail meat after enriching their diets with camelina or hempseed cakes.

The results of research conducted with chickens and the fact that quail belong to the same gallinaceous birds (*Galliformes* order) allow us to assume that camelina and hempseed cakes in their diets should have an effect on the fatty acid content changes in quail meat. The knowledge that camelina and hempseed cakes differ in their PUFA composition also leads to the assumption that cake feeding effects on the accumulation of fatty acids in quail meat could be different.

The purpose of our study was to investigate the effects of dietary hempseed or camelina cakes on the fatty acid profiles of intramuscular fat in the muscles of quails.

## 2. Materials and Methods

### 2.1. Poultry, Management and Study Design

The study was carried out on the experimental farm of the LUHS Animal Science Institute.

The investigations were conducted in accordance with the law of the Republic of Lithuania for animal welfare and handling, Law No. IX-2271 [36], and a sub-statutory act by the State Food and Veterinary Service of Lithuanian Republic regarding the confirmation of the order on the animals for experiments, research, storage, maintenance and operating requirements [37].

One-day old quail males of the Estonian breed were obtained from a commercial hatchery. This breed was developed from the Japanese quail (*Coturnix coturnix japonica*) in Estonia and recognized in 1987. Three treatment groups of 63 birds each were formed from 189 birds in total. The groups consisted of 9 replicates of 7 birds each. The quail in the control group (C) were fed compound feed containing 10% rapeseed cake. In the second and third quail groups, rapeseed cake was replaced by 10% hempseed (HE) and camelina cakes (CA), respectively. The feed for all groups was isoprotein and isocaloric, produced according to the requirements of the NRC (National Research Council, 1994) (Table 1).

The composition of fatty acid in the feed for the meat type quail is presented in Table 2 and Table 3.

During the experimental period, water and feed were provided ad libitum. The quail were raised in “Comfortplast” brooders for up to 28 days, and each bird was provided with 145 cm^2^ floor space. From the age of 29 days, the quail were reared in “Comfortplast” cages. Each bird was provided with 225 cm^2^ of floor space. All birds were reared under the same conditions. The quail were individually weighed at the beginning, day 28 and day 42 of the study. Feed intake was measured daily per cage.

The temperature of 34–35 °C was maintained in the cages in the first week, 30 °C in the second week, 26 °C in the third week and 20 °C in the last week of raising. The light–dark schedules were L24:D0h and L23:D1h from day 1 to day 14 and day 15 to day 42, respectively. The temperature in the quail raising room was maintained at 20 °C, relative humidity amounted to 60–65%, air speed 0.3 m/s and ammonia concentration to 5 ppm.

The length of the study was 42 days. At the end of the study, a control slaughter was performed by selecting 15 quail from each group. Prior to slaughter, the birds had not been fed for 12 h and afterwards were slaughtered in a commercial EU-licensed abattoir. The quail were electrically stunned in a water bath and then had their jugular vein cut. Carcasses were anatomically dissected according to the methodological recommendation [38].

At slaughter, breast and leg muscle samples were taken from each bird for the fatty acid composition analysis. The musculus pectoralis major was taken for analysis from the breast. All the thigh and drumstick muscles were taken from the legs of the quail for analysis. The composition of fatty acids was also analyzed in the feed for over 28 days old quail in the control and experimental groups.

### 2.2. Fatty Acid Analyses

The lipids for fatty acids analysis were extracted using a chloroform (Chromasolv Plus for HPLC with 0.5–1.0% ethanol)/methanol (Chromasolv for HPLC ≥ 99.9%) mixture (2:1 *v*/*v*) following the method outlined by Folch et al. [39]. The preparation of fatty acid methyl esters (FAME) from the total lipids followed the protocol described by Christopherson and Glass [40]. The analysis of the FAME was conducted using a Shimadzu GC-2010 Plus gas chromatograph (Shimadzu Corp., Kyoto, Japan) equipped with a flame ionization detector. The separation of FAME was carried out on a capillary column Rt-2560 (100 m; 0.25 mm ID; 0.25 µm df) from Restek (Bellefonte, PA, USA) through temperature programming ranging from 160 °C to 230 °C.

The injector temperature was maintained at +240 °C and the detector at +260 °C. The carrier gas (nitrogen) flow rate was set at 0.79 mL/min. The total duration of the gas chromatography analysis was 60 min, and the injection volume was 1.0 µL. The identification of FAME was achieved by comparing its retention times with those of authentic standard mixtures (Supelco 37 comp. FAME mix.) The relative proportion of each fatty acid in the sample was expressed as a percentage relative to the total sum of fatty acids using the ”LABSolutions LC/GC” (version 5.71) software for Shimadzu gas chromatograph workstations.

### 2.3. Lipid Quality Indices

The atherogenic index (AI) and the thrombogenic index (TI) [16,41], hypocholesterolemic/hypercholesterolemic (h/H) [42], peroxidability index (PI) [43] and the desirable fatty acids (DFA) [44] and hypercholesterolemic saturated fatty acids (HSFA) [45] indices were calculated on the basis of fatty acid analysis data.

### 2.4. Cholesterol Determination

The cholesterol content in quail muscles was determined according to the method described by Polak et al. [46]. For that purpose, we used a Shimadzu (Shimadzu corp., Kyoto, Japan) low pressure gradient HPLC system, consisting of a solvent delivery module LC-10ATVP, auto injector SIL-10ADVP, column oven CTO-10ACVP, UV–Vis detector SPD-10AVVP, system controller SCL-10AVP and on-line degasser DGU-14A. For HPLC system control and data collection, we used a Workstation LC Solution (Shimadzu corp., Kyoto Japan). A LiChrospher 100 RP-18e (150 × 4.6 mm, 5 μm) chromatography column (Alltech Associates Inc., Deerfield, Illinois, USA) was used for cholesterol separation. The chromatogram was executed at a wavelength of 210 nm. The mobile phase consisted of acetonitrile and 2-propanol (55:45), and a flow rate of 1.0 mL min^−1^ was used.

### 2.5. Statistical Analyses

Descriptive statistics were used to analyze the results of our study. Statistical analysis was conducted using the software package Statistica (StatSoft Inc. STATISTICA 2006, Version 7). The results are presented as mean values and standard error (SE). Statistical data were examined through a standard one-way analysis of variance (ANOVA) methods. In instances where ANOVA revealed differences between the groups, the significance of these differences within specific groups were determined using the Ryan–Einot–Gabriel–Welsh studentized range Q (REGWQ) post hoc test. The differences were considered statistically significant when *p* < 0.05.

## 3. Results and Discussion

### 3.1. Growth Performance

Our study indicated that the addition of hempseed cake had negatively affected the growth performance of quail (Table 4). The live weights of HE quail at the age of 28 and 42 days were, respectively, 9.83 g (*p* < 0.01) and 12.01 g (*p* < 0.01) lower in comparison with C group. Feed intake in the HE quail group was 19 g lower throughout the entire raising period (*p* < 0.05). This led to an increase in feed conversion ratio (FCR) (*p* < 0.05).

The reason for the decrease in quail growth and feed intake in the HE group can be explained by a higher crude fiber content in hempseed cake, which could have reduced nutrient digestibility [47]. Similar data were obtained in a study with chickens by Stastnik et al. [31].

Meanwhile, the growth performance parameters of CA quail did not differ from the control group. Other researchers have also found that camelina cake does not have a negative effect on the growth performance of chickens [10] and quail [48,49].

### 3.2. Carcass Dissection Data

Our study showed that the use of hempseed and camelina cakes in the diet of quail did not affect anatomical carcass dissection indicators (Table 5). No differences were found between the groups for the carcass yield and non-edible internal, heart, liver and abdominal fat. The proportions of the most valuable breast and leg muscles in the carcass did not decrease either (Table 5). No changes in anatomical carcass dissection data were found by Bulbul et al. [48] and Konca et al. [50], who also used camelina or hempseed in quail feed.

### 3.3. Fatty Acid Profiles of Intramuscular Fat in the Muscles of Quail

#### 3.3.1. Saturated Fatty Acids

Our study showed that supplementing quail diets with hempseed or camelina cakes did not increase the total SFA content in quail meat; however, differences between individual saturated fatty acids were found (Table 6 and Table 7).

In our study, lauric acid (C12:0), which is most harmful to humans, was not detected in CA-treated quail leg meat [51]. However, it was found (0.01%) in the control and HE groups. This is in agreement with the studies of other authors who also indicated that lauric acid (C12:0) was not detected in the meat of CA-treated chickens [7,8,10,11,33,34].

Conversely, the content of less harmful to human’s palmitic acid (C16:0) was found to be higher in the breast and leg meat of CA-treated quail. Jaskiewicz et al. [52] who studied camelina oil addition to chicken feed also indicated an increased content of palmitic (C16:0) acid in chicken breast and leg muscles.

Moreover, a higher content of arachidic (C20:0) acid was detected in the leg meat of both CA- and HE-treated quail (*p* < 0.05–*p* < 0.01). Arachidic (C20:0) acid in human food has been shown to improve their blood lipid profiles [53]. Other researchers also found more arachidic (C20:0) acid in chickens fed camelina supplement [10].

#### 3.3.2. Monosaturated Fatty Acids

The CA group quail received more monounsaturated fatty acids (MUFA) with their feed and their meat also had higher amounts of MUFA in comparison with the control group (*p* < 0.05).

The amount of dominant oleic acid (C18:1n-9) in CA quail meat was higher (*p* < 0.05) compared to the control group. Leg meat contained 1.2 times more oleic acid (C18:1n-9) than breast. Since oleic acid (C18:1n-9) affects the reduction in LDL-cholesterol in the human body [9,54], CA group quail meat is more favorable for human health due to the higher content of this acid.

Eicosenoic acid (C20:1n-9) in the fatty acid profile of quail meat increased more than three times compared to the control group (*p* < 0.01). Such changes can be explained by the fact that the feed offered to the CA group contained 10 times more eicosenoic acid (C20:1n-9).

A higher amount (*p* < 0.01) of erucic acid (C22:1n-9) was also detected in CA meat, but it was lower than the European Union limit for human consumption (5%) [55].

Studies with other poultry species such as chickens and ducks indicated that the supplementation of the diets with camelina cake lowered the total MUFA content in the tissues [10,26,56]. It is claimed that the feed with a higher n-3 PUFA content inhibits ∆9-desaturase enzyme activity, which reduces MUFA synthesis in the body [57,58]. Meanwhile, in our study, an increase in MUFA content in quail meat was found. Different results could have been caused by the peculiarities of quail physiology and the fact that their feed conversion ratio is higher than that of other bird species [5], and it could be assumed that the increase in n-3 PUFA in the feed was not sufficient to inhibit MUFA synthesis.

Enriching the diet of quail with camelina cake increased the accumulation of MUFA in the meat making it more useful for humans because MUFA reduces LDL-cholesterol levels [51], and, consequently, a lower cholesterol level reduces the risk of cardiovascular disease [59] and increases the anti-carcinogenic effect [60].

Meanwhile, hempseed cake addition to the quail feed had no influence on the MUFA content profile in quail meat.

#### 3.3.3. Polyunsaturated Fatty Acids

n-6 PUFA: In our study, supplementation of quail diets with camelina cake resulted in 1.2 times lower content of total n-6 PUFA. This had a direct influence on the FA changes in meat: the amounts of linoleic (C18:2 n-6; LA) (*p* < 0.01), arachidonic (C20:4n-6) (*p* < 0.01) acids, total n-6 PUFA (*p* < 0.01) and LC n-6 PUFA (*p* < 0.05) decreased in meat (Table 8 and Table 9).

Meanwhile, the results of research with other bird species are controversial. Some studies showed that the use of camelina cake influenced the increase in LA accumulation [8,10]; however, other authors did not report any effects [7,26,34].

The reason for this could be the assumption that higher dietary ALA causes competition for the same elongation–desaturation enzymes necessary for the synthesis of both n-3 and n-6 LC PUFA fatty acids, thus resulting in a lower LA content [61]. Meat with lower n-6 PUFA content is healthier, because n-6 PUFA in human food are promoters of inflammatory processes and increase the risk of developing cardiovascular diseases [51].

Our study showed that hempseed cake supplementation did not increase the total n-6 PUFA accumulation in quail muscles. However, an increase in the amount of γ-linolenic acid (C18:3n-6; GLA) was found in the meat of HE group (*p* < 0.01). Jing et al. [35] also found an increase in this acid in chicken meat after the trials with hemp oil. GLA has anti-inflammatory and anti-proliferative properties. Food containing GLA is a preventive measure against coronary heart disease, rheumatoid arthritis and ensures skin health [62,63,64,65].

n-3 PUFA: In our study, the addition of camelina or hemp cakes to quail feed increased the amount of n-3 PUFA in their meat. However, changes in the n-3 PUFA profile were more significant with camelina cake than hempseed cake addition (Table 8 and Table 9).

The ALA content in the feed for the CA group was 3.4 times higher than in the control and this resulted in 2.5 times higher ALA content in the breast and leg meat of this group (*p* < 0.01). Aziza et al. [7] also reported similar changes in the breast and leg meat of chickens fed the diet with 10% camelina cake.

Meanwhile, supplementing the diet with hempseed cake resulted in a 1.2-fold ALA increase in the feed compared to the control. The ALA content in the breast and leg meat of HE group was 1.3 times higher (*p* < 0.01), but the accumulation was lower compared to the CA group. Yalcin et al. [66] in the trial with hempseed (5–20%) addition to the feed have also reported ALA increase in quail breast meat. A higher content of ALA was also found in the breast and thigh meat of chickens fed hemp oil (3–6%)-supplemented diets [35].

However, in our study, no LC n-3 PUFA increase was observed in HE-treated quail muscle tissues. This is in agreement with the study of Skrivan et al. [67], who found no differences in LC n-3 PUFA content in chickens fed 4% hempseed. Conversely, Jing et al. [35], in a trial with chickens given hempseed oil, reported an increase in all individual LC n-3 PUFA and total LC n-3 PUFA in the meat. This result was determined by the use of hempseed oil (3–6%), which contains more fatty acids than seed or cake.

In our trial with quail, the diet enriched with camelina cake had influenced the bioconversion of LC n-3 PUFA from ALA. The muscle tissues of CA-treated quail contained 3–3.4 times more eicosapentaenoic (C20:5n-3; EPA) (*p* < 0.01), 1.2 times more docosapentaenoic (C22:5n-3; DPA) (*p* < 0.05–*p* < 0.01) and 1.3 times more docosahexaenoic (C22:6n-3; DHA) (*p* < 0.01) acid compared with the control group, and this resulted in the increase in total LC n-3 PUFA (*p* < 0.01).

The breast meat of CA-treated quail contained an amount of DHA similar to that determined by Aronen et al. [33] in their study with chickens given 25% camelina cake. Meanwhile, DHA accumulation in quail leg muscles was higher than that indicated by Aziza et al. and Nain et al. [7,27] in their trials with chickens fed camelina cake-supplemented diets.

Other authors who used camelina cake in chicken diets found an increase in only single LC n-3 PUFA in the muscles, although in their studies the content of ALA in the feed was higher (17.8%) [7,27,33] than in our study (13.43%). Thus, it can be assumed that augmentation of n-3 PUFA in the diet triggers the synthesis of LC n-3 PUFA from ALA in the body of quails, like in other birds, which is perhaps even more intense than in chickens and ducks.

The above changes in the amounts of n-3 PUFA resulted in an increase in total n-3 PUFA (*p* < 0.01) in CA-treated quail meat. An increase in total n-3 PUFA in the muscles of chickens fed camelina cake-supplemented diets was also observed by Aronen et al., Aziza et al., Nain et al., Orczewska-Dudek and Pietras and Ryhänen et al. [7,10,27,33,34].

However, the amount of total n-3 PUFA was higher in the breast and leg meat of CA-treated quail than that reported by Aziza et al. [7] in the study with chickens (4.64–5.48 vs. 3.33–4.27%) and in breast meat it was also higher than that found by Juodka et al. [26] in the study with ducklings (5.48 vs. 4.53%).

The use of camelina cake in the diet of quail reduced the n-6/n-3 PUFA ratio in the CA diet and meat by 4.23 and 2.10–2.39 times, respectively, compared to the control group (Table 8 and Table 9). In CA-treated quail meat, this ratio approached the recommended (5.17–5.74 vs. 4–5) [68]. Meanwhile, the addition of hempseed cake to the diet reduced the n-6/n-3 PUFA ratio in quail meat to only 21.85–22.53.

The addition of camelina cake to the quail diet had greater influence on the n-3 PUFA profile changes than addition of hempseed cake. Research has shown and this is in agreement with our study that the lower is the linoleic/α-linolenic (LA/ALA) ratio in the diet, the higher is the expression of genes (FADS1, FADS2, ELOVL2 and ELOVL5) related with lipid metabolism in the liver and breast of broiler chickens [69,70]. The use of hempseed cake in the quail diet resulted in the LA/ALA ratio being 12.88, whereas the addition of camelina cake reduced it to 3.74 and determined a higher n-3 PUFA accumulation in the muscle tissues. The LA/ALA ratio in the muscles of CA-treated quail decreased to 8.46–9.38 and the LC n-6/LC n-3 ratio to 1.94–3.22, while in HE group the LA/ALA ratio was 2.5 times higher.

Although the use of camelina and hempseed cakes showed different efficacies of n-3 PUFA accumulation in the muscles, the effect was significant and allowed the production of quail meat of exceptional quality.

The amount of EPA + DHA in the breast meat of HE and CA-treated quail was, respectively, 50.12 and 66.64 mg/100 g, whereas in the leg meat, this amount was, respectively, 52.60 and 69.58 mg/100 g. The amount of EPA + DHA in the breast and leg meat of CA-treated quail was from 1.33 to 1.32 times higher than that in the HE-treated group. In accordance with the European Commission Nutrition Claims, the meat of CA and H treated quail can be labeled as “a source of omega-3 fatty acids” [71].

In the human body, the synthesis of individual LC n-3 PUFA from dietary ALA is limited and accounts to 1% in infants [72] and to 0.05–0.2% in adults [73]. A sufficient LC n-3 PUFA amount in human nutrition is a factor that reduces the occurrence of cardiovascular disease and Alzheimer’s disease and increases anti-inflammatory effects in the human body [51].

#### 3.3.4. Indices

The atherogenic index (AI) is the ratio between pro-atherogenic and anti-atherogenic fatty acids [74]. The value of AI in our study did not change in the HE group, while in the CA group it was higher (*p* < 0.05–*p* < 0.01) in the breast and leg meat compared to the C group and reached 0.3 (Table 10 and Table 11). However, this indicator is more favorable than that found in the meat of other bird species, i.e., in chicken (0.49–0.63) [15,75,76], turkey (0.47) [77], geese (0.29–0.37) [78,79] and duck (0.35–0.40) [80] without using any feed additives.

In our study, the AI indicators of quail meat were higher than those found in free-range chickens [76]. In our previous study, a higher AI was obtained in the leg meat of ducks given camelina cake than in quail [26]. When AI is lower than 0.5, the possibility of developing coronary diseases decreases [74,81].

Thrombogenic index (TI), shows the possibility of clot formation in the blood vessels. The recommended value of this index in human food should be less than 1 [74,81].

Our research showed that the CA-treated quail meat had the lowest TI (0.54–0.60) (*p* < 0.05–*p* < 0.01).

This index in the breast meat of CA-treated quail was lower than those found in other studies with chicken (0.94–1.27) [15,75,76], turkey (0.63–0.91) [77,82], geese (0.61–0.74) [78,79] and duck (0.83–0.98) [80] offered standard feed and ducks fed camelina cake supplement [26].

Our results show that the AI and TI indices of quail meat are lower than those of other bird species, which makes it possible to assert that, according to these indices, the meat of quail fed both conventionally and with hempseed or camelina supplements is the healthiest of all poultry species. Such quail meat can protect against coronary artery disease.

h/H index indicate the ratio between hypocholesterolemic and hypercholesterolemic fatty acids and the higher the ratio, the healthier is the food product. An h/H index score of higher than 2 reduces the risk of developing cardiovascular diseases, including atherosclerosis, because it lowers the level of LDL cholesterol in blood serum [83,84,85,86,87,88].

The highest h/H index in our study were found in the leg meat of HE-treated quail, and they were higher than those in the C group (*p* < 0.05). The lowest h/H index was found in the CA group, lower than that in the C group (*p* < 0.01).

Our research showed that the value of h/H index in quail meat was higher and more favorable to human health than that found in other studies with turkeys (1.26–1.41) [82], chickens (1.21–2.61) [15,75,89] and geese (2.60–3.47) [78,90] given standard feed and with ducklings fed rapeseed, hempseed or camelina-supplemented diets (2.45–2.88) [26].

Desirable fatty acid (DFA) and hypercholesterolemic saturated fatty acid (HSFA) indicators did not differ between HE and C quail groups. CA muscles showed lower DFA and higher HSFA (*p* < 0.05–*p* < 0.01) contents compared to C. In our study, there were no significant differences between the groups for the cholesterol levels in both breast and leg meat. In the C and CA groups, more cholesterol was found in the leg than breast meat. The cholesterol level in the breast meat of the quail in our study was lower than that indicated by Quaresma et al. [13] in their study of farmed Japanese quail (71.07 mg) and wild Common quail (64.38 mg). Pietras and Orczewska-Dudek [11] also did not find any difference in cholesterol levels between the groups in their study with chickens fed a camelina oil-supplemented diet.

## 4. Conclusions

The supplementation of diets with oil industry by-products rich in n-3 PUFA had a significant impact on the changes in the intramuscular fatty acid profile of quail.

The enrichment of quail diet with hempseed cake (10%) increased the amounts of beneficial to humans GLA and ALA and total n-3 PUFA and decreased n-6/n-3 PUFA and LA/ALA ratios in intramuscular fat compared to C group quail fed rapeseed cake. However, the effect of hempseed cake on the accumulation of ALA and its bioconversion to LC n-3 PUFA was lower compared to the effect of camelina cake addition. Meanwhile, hempseed cake supplementation had a negative effect on growth performance, also resulting in lower feed intake and higher FCR. The use of camelina cake (10%) supplement in a quail diet did not show any negative impact on growth performance and increased the amounts of MUFA and total n-3 PUFA. The accumulation of ALA and LC n-3 PUFA was 2.5 and 1.4 times higher in comparison with that of the C group quail meat. The decrease was observed in the amounts of LA, total n-6 PUFA, n-6/n-3 PUFA and LA/ALA ratios and TI index.

Enriching the diet of quail with camelina cake makes it possible to produce meat with a lower n-6/n-3 PUFA ratio and provide consumers with health-beneficial products.

Meanwhile, the addition of hempseed cake made it possible to produce quail meat of exceptional quality with an increased GLA content. A wider employment of oil industry by-products in quail farming could contribute to higher production sustainability and reduce dependence on imported feed materials.

## Figures and Tables

**Table 1:** Diet composition used in the trial.

Specification	Control Group (C)		Experimental 1 (HE)		Experimental 2 (CA)	
	Until 28 Days	Over 28 Days	Until 28 Days	Over 28 Days	Until 28 Days	Over 28 Days
Wheat, %	19.93	31.76	18.48	29.84	20.67	32.74
Barley, %	4.00	6.00	4.00	6.00	4.00	6.00
Maize, %	20.00	20.00	20.00	20.00	20.00	20.00
Peas, %	8.00	10.00	8.00	10.00	8.00	10.00
Sunflower meal, %	10.00	8.61	10.00	10.60	10.00	7.63
Rape cake, %	10.00	10.00	0.00	0.00	0.00	0.00
Hempseed cake, %	0.00	0.00	10.00	10.00	0.00	0.00
Camelina cake, %	0.00	0.00	0.00	0.00	10.00	10.00
Soybean meal, %	18.07	4.00	19.56	4.00	17.34	4.00
Brewers’ yeast, %	5.00	5.00	5.00	5.00	5.00	5.00
Oyster shells, %	0.5	0.5	0.5	0.5	0.5	0.5
Feeder’s chalk, %	1.24	1.25	1.39	1.43	1.25	1.27
Premix, % *	1.00	1.00	1.00	1.00	1.00	1.00
Sunflower oil, %	1.50	1.00	1.50	1.00	1.50	1.00
Fodder salt, %	0.1	0.1	0.1	0.1	0.1	0.1
Monocalcium phosphate, %	0.66	0.78	0.47	0.53	0.64	0.76
Calculated nutritional value of feed mixture						
Dry matter, kg/kg	0.87	0.87	0.87	0.87	0.87	0.87
Metabolizable energy, MJ/kg	10.93	11.20	11.17	11.39	11.03	11.32
Crude protein, g/kg	240.04	190.51	240.03	190.51	240.03	190.50
Lysine, g/kg	12.62	9.24	12.24	8.71	12.28	8.98
Methionine + Cysteine, g/kg	6.96	5.88	6.96	6.05	7.32	6.16
Threonine, g/kg	8.92	7.23	8.53	6.87	8.74	7.02
Tryptophan, g/kg	2.81	2.33	2.63	2.17	2.78	2.28
Fiber, g/kg	59.39	49.89	75.00	70.53	60.96	50.27
Fat, g/kg	47.39	42.59	40.96	36.59	42.16	37.15
Calcium, g/kg	12.48	12.37	12.46	12.39	12.46	12.38
Phosphorus, g/kg	7.23	7.02	7.30	7.09	7.28	7.01
Glucosinolates, g/kg	0.16	0.16	0.00	0.00	0.25	0.25

* The composition premix per kilogram: retinol—640 international units; α—tocopherol acetate—3 mg; thiamine—6.6 mg; riboflavin—19.9 mg; pyridoxine—13.2 mg; cobalamin—0.02 mg; biotin—0.88 mg; nicotinic acid—226.4 mg; pantothenic acid—17.7 mg; folic acid—8.8 mg; choline—2.3 mg; calcium—195 g; phosphorus—6.3 g; sodium—0.34 g; iron—153.6 mg; zinc—44.1 mg; lysine—17.9 g; methionine—3.8 g; cysteine—1.8 g; threonine—11.8 g; tryptophan—2.2 g; isoleucine—11 g; tyrosine—21 g; phenylalanine—23.4 g; valine—11.7 g; histidine—8.6 g; arginine—12.9 g; proline—4.5 g; asparagine—21 g; leucine—15 g.

**Table 2:** Nutritional value and fatty acid composition of rapeseed, hempseed and camelina cakes.

Item	Rapeseed Cake	Hempseed Cake	Camelina Cake
Dry matter %	88.86	94.88	89.42
Metabolizable energy MJ/kg	8.24	11.01	9.03
Crude protein %	36.12	30.95	38.61
Lysine g/kg	17.80	10.30	16.20
Methionine + cysteine g/kg	10.90	9.90	15.00
Threonine g/kg	14.50	8.80	13.60
Tryptophan g/kg	4.30	2.00	4.20
Ether extract %	12.71	6.30	7.46
Crude fiber %	12.35	27.32	14.23
Calcium %	0.58	0.25	0.57
Phosphorus %	0.95	1.42	1.07
Glucosinolates %	0.16	0.00	0.25
Fatty acids (% of total fatty acids):			
Capric (C10:0)	0.00	0.04	0.00
Myristic (C14:0)	0.13	0.05	0.06
Pentadecanoic (C15:0)	0.10	0.04	0.04
Palmitic (C16:0)	8.22	6.34	6.12
Margaric (C17:0)	0.07	0.05	0.04
Stearic (C18:0)	1.36	2.35	2.17
Arachidic (C20:0)	0.36	0.70	1.28
Behenic (C22:0)	0.20	0.32	0.33
Lignoceric (C24:0)	0.16	0.12	0.23
SFA *	10.60	10.01	10.27
Palmitoleic (C16:1n-9)	1.13	0.02	0.05
Hexadecenoic (C16:1n-7)	0.10	0.10	0.13
Heptadecenoic (C17:1n-9)	0.17	0.02	0.00
Elaidic (C18:1n-9t)	0.31	0.00	0.22
Oleic (C18:1n-9)	41.34	8.84	12.57
Vaccenic (C18:1n-7)	10.55	0.91	1.33
Eicosenoic (C20:1n-9)	0.66	0.35	11.73
Erucic (C22:1n-9)	0.21	0.00	2.52
Nervonic (C24:1)	0.00	0.06	1.16
MUFA *	54.47	10.30	29.71
Octadecadienoic (C18:2n-6c,t)	0.33	0.07	0.00
Linoleic (C18:2n-6)	28.42	57.26	20.65
γ-linolenic (C18:3n-6)	0.06	1.99	0.11
Eicosadienoic (C20:2n-6)	0.00	0.80	1.85
Docosadienoic (C22:2n-6)	0.00	0.00	0.40
n-6 PUFA	28.81	60.12	23.01
α-linolenic (C18:3n-3)	6.12	19.51	35.54
Eicosatrienoic (C20:3n-3)	0.00	0.06	1.47
n-3 PUFA	6.12	19.57	37.01
PUFA *	34.93	79.69	60.02
n-6/n-3 PUFA ratio	4.71	3.07	0.62
linoleic (C18:2)/α-linolenic (C18:3n-3) ratio	4.64	2.93	0.58

* SFA—saturated fatty acids; * MUFA—monounsaturated fatty acids; * PUFA—polyunsaturated fatty acids.

**Table 3:** Composition of fatty acid content in the feed over 28 days old quail in control and experimental groups (% of total fatty acids).

Fatty Acid	Control (C)	Experimental 1 (HE)	Experimental 2 (CA)
Capric (C10:0)	0.05	0.01	0.03
Myristic (C14:0)	0.07	0.06	0.07
Palmitic (C16:0)	9.05	8.75	9.68
Margaric (C17:0)	0.05	0.04	0.00
Stearic (C18:0)	2.50	2.51	2.26
Arachidic (C20:0)	0.32	0.35	0.63
Behenic (C22:0)	0.37	0.38	0.29
Lignoceric (C24:0)	0.17	0.19	0.20
SFA *	12.58	12.29	13.16
Oleic (C18:1n-9)	17.08	16.07	15.46
Eicosenoic (C20:1n-9)	0.33	0.37	3.34
Erucic (C22:1n-9)	0.04	0.00	0.73
MUFA *	19.27	17.80	21.13
Octadecadienoic (C18:2n-6c, t)	0.06	0.05	0.00
Linoleic (C18:2n-6)	63.11	63.88	50.19
γ –linolenic (C18:3n-6)	0.36	0.53	0.50
Eicosadienoic (C20:2n-6)	0.11	0.16	0.71
Arachidonic (C20:4n-6)	0.46	0.21	0.39
Docosadienoic (C22:2n-6)	0.01	0.11	0.10
n-6 PUFA	64.11	64.94	51.89
α-linolenic (C18:3n-3)	4.01	4.96	13.43
Eicosatrienoic (C20:3n-3)	0.03	0.01	0.39
n-3 PUFA	4.04	4.97	13.82
PUFA *	68.15	69.91	65.71
n-6/n-3 PUFA ratio	15.87	13.07	3.75
Linoleic/α-linolenic ratio	15.74	12.88	3.74

* SFA—saturated fatty acids; * MUFA—monounsaturated fatty acids; * PUFA—polyunsaturated fatty acids.

**Table 4:** Effect of dietary hempseed or camelina cakes on growth performance of quails.

Item	Group					
	Control (C)		Experimental 1 (HE)		Experimental 2 (CA)	
	Mean	SE	Mean	SE	Mean	SE
Live weight, g						
28 days	165.56 ^A^	1.945	155.73 ^B^	1.737	161.64	1.694
42 days	214.01 ^A^	2.624	202.00 ^B^	2.371	208.40	2.547
Feed intake (g/bird)						
1–28 day	277.00	3.873	267.57	3.022	273.71	3.205
29–42 day	288.29	4.224	278.00	2.370	281.43	3.108
1–42 day	565.29 ^a^	6.998	545.57 ^b^	3.265	555.14	5.152
Feed conversion ratio						
1–28 day	1.77 ^B^	0.004	1.82 ^A^	0.018	1.79	0.013
29–42 day	5.97	0.124	6.02	0.079	6.03	0.085
1–42 day	2.75 ^b^	0.012	2.82 ^a^	0.025	2.78	0.020

(C)—diet with 10% rapeseed cake; Experimental 1 (HE)—diet with 10% hempseed cake; Experimental 2 (CA)—diet with 10% camelina cake; and SE—standard error. ^a, b^—means in the same row with different superscripts differ significantly at *p* < 0.05. ^A, B^—means in the same row with different superscripts differ significantly at *p* < 0.01.

**Table 5:** Effect of dietary hempseed or camelina cakes on quail carcass dissection data (%).

Item	Group					
	Control (C)		Experimental 1 (HE)		Experimental 2 (CA)	
	Mean	SE	Mean	SE	Mean	SE
Carcass yield	71.31	2.174	72.44	0.820	71.71	0.460
Non-edible internal parts	9.84	0.930	7.80	0.467	8.72	0.672
Heart	1.21	0.037	1.25	0.039	1.19	0.034
Gizzard	2.34 ^A^	0.116	2.08	0.080	1.95 ^B^	0.085
Liver	2.18	0.140	2.34	0.089	2.35	0.233
Abdominal fat	2.44	0.373	2.76	0.459	1.96	0.286
Breast muscle	22.40	3.194	22.49	1.795	22.33	2.796
Leg muscle	18.29	2.099	19.86	0.777	19.50	0.851

Control (C)—diet with 10% rapeseed cake; Experimental 1 (HE)—diet with 10% hempseed cake; Experimental 2 (CA)—diet with 10% camelina cake; SE—standard error; SFA (saturated fatty acid); MUFA (monounsaturated fatty acid). ^A, B^—means in the same row with different superscripts differ significantly at *p* < 0.01.

**Table 6:** Saturated and monounsaturated fatty acid in the breast (*m. pectoralis major*) (% of total fatty acids).

Fatty Acid	Group					
	Control (C)		Experimental 1 (HE)		Experimental 2 (CA)	
	Mean	SE	Mean	SE	Mean	SE
Lauric (C12:0)	0.01	0.003	0.01	0.003	0.01	0.003
Myristic (C14:0)	0.34 ^b^	0.008	0.33 ^b^	0.010	0.37 ^a^	0.010
Pentadecanoic (C15:0)	0.04 ^A^	0.001	0.03 ^B,b^	0.001	0.04 ^A,a^	0.001
Palmitic (C16:0)	18.13 ^B^	0.158	18.10 ^B,b^	0.277	19.12 ^A,a^	0.275
Margaric (C17:0)	0.09	0.004	0.08	0.003	0.09	0.005
Stearic (C18:0)	9.79	0.261	9.49	0.238	9.53	0.264
Arachidic (C20:0)	0.06	0.002	0.06	0.002	0.07	0.002
Behenic (C22:0)	0.06 ^A^	0.005	0.03 ^B^	0.002	0.08 ^A^	0.011
Total SFA	28.52 ^a,b^	0.374	28.14 ^b^	0.414	29.30 ^a^	0.323
Myristoleic (C14:1n-7)	0.09	0.007	0.08	0.007	0.09	0.006
Palmitelaidic (C16:1n-7t)	0.01	0.003	0.01	0.003	0.01	0.004
Hexadecenoic (C16:1n-9)	0.25	0.007	0.26	0.011	0.27	0.006
Palmitoleic (C16:1n-7)	4.88	0.304	4.61	0.312	4.98	0.277
Heptadecenoic (C17:1n-9)	0.05 ^A^	0.003	0.03 ^B^	0.004	0.05 ^A^	0.004
Elaidic (C18:1n-9t)	0.07	0.003	0.07	0.002	0.08	0.010
Oleic (C18:1n-9)	24.53 ^b^	0.514	25.69 ^a,b^	0.597	26.83 ^a^	0.811
Vaccenic (C18:1n-7)	1.87	0.027	1.79	0.056	1.92	0.063
Eicosenoic (C20:1n-9)	0.19 ^B^	0.005	0.20 ^B^	0.006	0.57 ^A^	0.024
Erucic (C22:1n-9)	0.00 ^B^	0.000	0.00 ^B^	0.000	0.03 ^A^	0.005
Total MUFA	31.93 ^b^	0.786	32.74 ^a,b^	0.717	34.82 ^a^	1.070

Control (C)—diet with 10% rapeseed cake; Experimental 1 (HE)—diet with 10% hempseed cake; Experimental 2 (CA)—diet with 10% camelina cake; SE—standard error; SFA (saturated fatty acid); MUFA (monounsaturated fatty acid). ^a, b^—means in the same row with different superscripts differ significantly at *p* < 0.05. ^A, B^—means in the same row with different superscripts differ significantly at *p* < 0.01.

**Table 7:** Saturated and monounsaturated fatty acid in the leg muscle (% of total fatty acids).

Fatty Acid	Group					
	Control (C)		Experimental 1 (HE)		Experimental 2 (CA)	
	Mean	SE	Mean	SE	Mean	SE
Lauric (C12:0)	0.010 ^A^	0.003	0.010 ^a^	0.003	0.00 ^B,b^	0.000
Myristic (C14:0)	0.39 ^A,B^	0.008	0.38 ^B^	0.006	0.42 ^A^	0.009
Pentadecanoic (C15:0)	0.05 ^A^	0.001	0.04 ^B,b^	0.002	0.05 ^a^	0.001
Palmitic (C16:0)	17.45 ^b^	0.183	16.90 ^B^	0.271	18.27 ^A,a^	0.275
Margaric (C17:0)	0.08	0.004	0.08	0.003	0.08	0.004
Stearic (C18:0)	7.14	0.210	7.11	0.318	7.36	0.347
Arachidic (C20:0)	0.06 ^B,b^	0.002	0.08 ^a^	0.005	0.08 ^A^	0.004
Behenic (C22:0)	0.05 ^A^	0.004	0.03 ^B^	0.005	0.05 ^A^	0.005
Total SFA	25.22 ^a,b^	0.340	24.62 ^b^	0.475	26.31 ^a^	0.448
Myristoleic (C14:1n-7)	0.09 ^a,b^	0.006	0.07 ^b^	0.005	0.09 ^a^	0.006
Palmitelaidic (C16:1n-7t)	0.01	0.003	0.01	0.003	0.01	0.003
Hexadecenoic (C16:1n-9)	0.26	0.010	0.26	0.012	0.27	0.008
Palmitoleic (C16:1n-7)	5.12	0.284	4.69	0.261	5.44	0.356
Heptadecenoic (C17:1n-9)	0.04 ^B^	0.002	0.04 ^B^	0.004	0.06 ^A^	0.003
Elaidic (C18:1n-9t)	0.01	0.011	0.08	0.002	0.08	0.004
Oleic (C18:1n-9)	30.03 ^b^	0.463	30.76 ^a,b^	0.676	32.72 ^a^	1.014
Vaccenic (C18:1n-7)	1.80 ^A^	0.020	1.63 ^B^	0.045	1.85 ^A^	0.062
Eicosenoic (C20:1n-9)	0.23 ^B^	0.005	0.24 ^B^	0.007	0.79 ^A^	0.045
Erucic (C22:1n-9)	0.00 ^B^	0.000	0.00 ^B^	0.000	0.04 ^A^	0.003
Total MUFA	37.67 ^b^	0.691	37.78 ^b^	0.818	41.35 ^a^	1.314

Control (C)—diet with 10% rapeseed cake; Experimental 1 (HE)—diet with 10% hempseed cake; Experimental 2 (CA)—diet with 10% camelina cake; SE—standard error; SFA (saturated fatty acid); MUFA (monounsaturated fatty acid). ^a, b^—means in the same row with different superscripts differ significantly at *p* < 0.05. ^A, B^—means in the same row with different superscripts differ significantly at *p* < 0.01.

**Table 8:** Polyunsaturated fatty acid in the breast (*m. pectoralis major*) (% of total fatty acids).

Fatty Acid	Group					
	Control (C)		Experimental 1 (HE)		Experimental 2 (CA)	
	Mean	SE	Mean	SE	Mean	SE
Linolelaidic (C18:2n-6t)	0.03 ^A^	0.002	0.02 ^B^	0.003	0.03 ^A^	0.002
Octadecadienoic (C18:2n-6c,t)	0.02 ^A^	0.004	0.00 ^B^	0.000	0.023 ^A^	0.003
Octadecadienoic (C18:2n-6t,c)	0.01 ^B^	0.004	0.03 ^A^	0.001	0.00 ^C^	0.000
Linoleic (C18:2n-6)	25.79 ^A^	0.352	25.59 ^A^	0.372	21.00 ^B^	0.542
γ-linolenic (C18:3n-6)	0.14 ^B^	0.004	0.17 ^A^	0.007	0.13 ^B^	0.004
Eicosadienoic (C20:2n-6)	0.14 ^B^	0.005	0.16 ^B^	0.008	0.23 ^A^	0.011
Eicosatrienoic (C20:3n-6)	0.26 ^B^	0.009	0.26 ^B^	0.010	0.31 ^A^	0.013
Arachidonic (C20:4n-6)	7.52 ^A^	0.350	7.07 ^A,a^	0.258	6.28 ^B,b^	0.238
Docosatetraenoic (C22:4n-6)	0.23 ^A^	0.009	0.18 ^B^	0.009	0.14 ^C^	0.008
Total LC n-6 PUFA	8.15 ^a^	0.356	7.66 ^a,b^	0.264	6.96 ^b^	0.248
Total n-6 PUFA	34.14 ^A^	0.434	33.47 ^A^	0.492	28.14 ^B^	0.686
α-linolenic (C18:3n-3)	0.90 ^C^	0.031	1.15 ^B^	0.036	2.26 ^A^	0.090
Eicosatrienoic (C20:3n-3)	0.00 ^B^	0.000	0.00 ^B^	0.000	0.07 ^A^	0.004
Eicosapentaenoic (C20:5n-3)	0.11 ^B^	0.008	0.13 ^B^	0.008	0.37 ^A^	0.023
Docosapentaenoic (C22:5n-3)	0.56 ^B^	0.027	0.57 ^B^	0.025	0.69 ^A^	0.028
Docosahexaenoic (C22:6n-3)	1.59 ^B^	0.090	1.66 ^B^	0.064	2.08 ^A^	0.093
Total LC n-3 PUFA	2.27 ^B^	0.102	2.36 ^B^	0.079	3.22 ^A^	0.122
Total n-3 PUFA	3.17 ^C^	0.082	3.51 ^B^	0.091	5.48 ^A^	0.179
Total PUFA	37.31 ^A^	0.462	36.99 ^A^	0.546	33.63 ^B^	0.839
n-6/n-3 ratio	10.85 ^A^	0.261	9.89 ^B^	0.209	5.17 ^C^	0.094
Linoleic (C18:2n-6)/α-linolenic (C18:3n-3) ratio	29.07 ^A^	0.860	22.53 ^B^	0.660	9.377 ^C^	0.216
PUFA/SFA	1.31 ^A^	0.018	1.32 ^A^	0.027	1.15 ^B^	0.027
PUFA/MUFA	1.18 ^A^	0.044	1.14 ^A,a^	0.044	0.99 ^B,b^	0.050

Control (C)—diet with 10% rapeseed cake; Experimental 1 (HE)—diet with 10% hempseed cake; Experimental 2 (CA)—diet with 10% camelina cake; SE—standard error; LC (long chain); PUFA (polyunsaturated fatty acid). ^a, b^—means in the same row with different superscripts differ significantly at *p* < 0.05. ^A, B, C^—means in the same row with different superscripts differ significantly at *p* < 0.01.

**Table 9:** Polyunsaturated fatty acid in the leg muscle (% of total fatty acids).

Fatty Acid	Group					
	Control (C)		Experimental 1 (HE)		Experimental 2 (CA)	
	Mean	SE	Mean	SE	Mean	SE
Linolelaidic (C18:2n-6t)	0.03 ^a^	0.002	0.02 ^b^	0.002	0.04 ^a,b^	0.007
Octadecadienoic (C18:2n-6c,t)	0.03 ^A^	0.003	0.00 ^B^	0.000	0.04 ^A^	0.007
Octadecadienoic (C18:2n-6t,c)	0.00 ^B^	0.003	0.03 ^A^	0.002	0.00 ^B^	0.000
Linoleic (C18:2n-6)	29.03 ^A^	0.399	29.44 ^A^	0.540	22.60 ^B^	0.653
γ-linolenic (C18:3n-6)	0.13 ^B^	0.002	0.17 ^A^	0.006	0.13 ^B^	0.004
Eicosadienoic (C20:2n-6)	0.11 ^C^	0.003	0.13 ^B^	0.005	0.18 ^A^	0.011
Eicosatrienoic (C20:3n-6)	0.16 ^B^	0.006	0.15 ^B^	0.008	0.20 ^A^	0.014
Arachidonic (C20:4n-6)	3.51	0.161	3.27	0.234	3.03	0.200
Docosatetraenoic (C22:4n-6)	0.27 ^A^	0.014	0.24 ^A^	0.017	0.16 ^B^	0.013
Total LC n-6 PUFA	4.04	0.178	3.77	0.261	3.58	0.232
Total n-6 PUFA	33.27 ^A^	0.463	33.44 ^A^	0.579	26.38 ^B^	0.808
α-linolenic (C18:3n-3)	1.05 ^C^	0.029	1.36 ^B^	0.048	2.71 ^A^	0.116
Eicosatrienoic (C20:3n-3)	0.00 ^B^	0.000	0.00 ^B^	0.000	0.07 ^A^	0.005
Eicosapentaenoic (C20:5n-3)	0.05 ^B^	0.003	0.05 ^B^	0.004	0.15 ^A^	0.012
Docosapentaenoic (C22:5n-3)	0.38 ^b^	0.020	0.40 ^a,b^	0.028	0.47 ^a^	0.036
Docosahexaenoic (C22:6n-3)	0.97 ^b^	0.052	1.03 ^a,b^	0.073	1.25 ^a^	0.090
Total LC n-3 PUFA	1.40 ^B^	0.069	1.47 ^B^	0.100	1.94 ^A^	0.135
Total n-3 PUFA	2.45 ^C^	0.069	2.86 ^B^	0.085	4.64 ^A^	0.197
Total PUFA	35.72 ^A^	0.491	36.28 ^A^	0.628	31.02 ^B^	0.990
Total trans acid	0.17	0.013	0.14	0.005	0.16	0.011
n-6/n-3 ratio	13.69 ^A^	0.380	11.90 ^B^	0.310	5.74 ^C^	0.119
Linoleic (C18:2n-6)/α-linolenic (C18:3n-3) ratio	27.88 ^A^	0.896	21.85 ^B^	0.651	8.46 ^C^	0.211
PUFA/SFA	1.42 ^A^	0.025	1.48 ^A^	0.035	1.18 ^B^	0.035
PUFA/MUFA	0.96 ^A^	0.029	0.97 ^A^	0.041	0.77 ^B^	0.046

Control (C)—diet with 10% rapeseed cake; Experimental 1 (HE)—diet with 10% hempseed cake; Experimental 2 (CA)—diet with 10% camelina cake; SE—standard error; LC (long chain); PUFA (polyunsaturated fatty acid). ^a, b^—means in the same row with different superscripts differ significantly at *p* < 0.05. ^A, B, C^—means in the same row with different superscripts differ significantly at *p* < 0.01.

**Table 10:** Indices in the breast (*m. pectoralis major*) (% of total fatty acids).

Item	Group					
	Control (C)		Experimental 1 (HE)		Experimental 2 (CA)	
	Mean	SE	Mean	SE	Mean	SE
Atherogenic index	0.26 ^b^	0.004	0.25 ^B^	0.005	0.28 ^A,a^	0.005
Thrombogenic index	0.58 ^a^	0.010	0.55 ^a,b^	0.015	0.54 ^b^	0.011
Hypo/hypercholesterolemic index	3.78 ^B,b^	0.054	3.98 ^A,a^	0.072	3.50 ^C^	0.061
Peroxidation index	58.28	1.260	58.81	1.716	56.58	2.314
Unsaturation index	123.18	0.792	124.46	1.204	119.73	1.557
Desirable fatty acids	79.04 ^A^	0.219	79.21	0.279	77.98 ^B^	0.273
Hypercholesterolemic saturated fatty acids	19.51 ^B^	0.171	19.45	0.030	20.60 ^A^	0.299
Cholesterol mg/100 g	67.53	2.637	59.44	3.532	65.25	2.769
Total lipids, %	2.99	0.142	2.80	0.178	2.72	0.168

Control (C)—diet with 10% rapeseed cake; Experimental 1 (HE)—diet with 10% hempseed cake; Experimental 2 (CA)—diet with 10% camelina cake; SE—standard error. ^a, b^—means in the same row with different superscripts differ significantly at *p* < 0.05. ^A, B, C^—means in the same row with different superscripts differ significantly at *p* < 0.01.

**Table 11:** Indices in the leg muscle (% of total fatty acids).

Item	Group					
	Control (C)		Experimental 1 (HE)		Experimental 2 (CA)	
	Mean	SE	Mean	SE	Mean	SE
Atherogenic index	0.26 ^b^	0.004	0.25 ^B^	0.005	0.28 ^A,a^	0.005
Thrombogenic index	0.58 ^a^	0.010	0.55 ^a,b^	0.015	0.54 ^b^	0.011
Hypo/hypercholesterolemic index	3.78 ^B,b^	0.054	3.98 ^A,a^	0.072	3.50 ^C^	0.061
Peroxidation index	58.28	1.260	58.81	1.716	56.58	2.314
Unsaturation index	123.18	0.792	124.46	1.204	119.73	1.557
Desirable fatty acids	80.52 ^a^	0.216	81.17	0.268	79.73 ^b^	0.280
Hypercholesterolemic saturated fatty acids	19.04 ^b^	0.199	18.42	0.286	19.95 ^a^	0.295
Cholesterol mg/100 g	67.53	2.637	59.44	3.532	65.25	2.769
Total lipids, %	4.74	0.186	4.87	0.242	4.97	0.277

Control (C)—diet with 10% rapeseed cake; Experimental 1 (HE)—diet with 10% hempseed cake; Experimental 2 (CA)—diet with 10% camelina cake; SE—standard error. ^a, b^—means in the same row with different superscripts differ significantly at *p* < 0.05. ^A, B, C^—means in the same row with different superscripts differ significantly at *p* < 0.01.

## Data Availability

Data are contained within the article.
